# Identification and Time Series Analysis of PM_2.5_ and O_3_ Associated Health Risk Prevention and Control Areas

**DOI:** 10.3390/toxics13050356

**Published:** 2025-04-29

**Authors:** Xinyu Huang, Bin Zou, Shenxin Li

**Affiliations:** School of Geosciences and Info-Physics, Central South University, Changsha 410083, China; 245011028@csu.edu.cn (X.H.); 210010@csu.edu.cn (B.Z.)

**Keywords:** air pollution, atmospheric remote sensing, health risk assessment, health equity, health GIS

## Abstract

Air pollution of PM_2.5_ and O_3_ is a global health concern. Traditional approaches for identifying air pollution control areas mainly relied on pollutant concentrations, neglecting population distribution and exposure. This study proposes a method to divide these areas from a health risk perspective, comparing their objectivity and rationality with the government-defined key regions. The results show that for PM_2.5_, the health risk population and average risk rates in the prevention and control areas were 0.993 million (0.1286%), 1.030 million (0.1283%), and 1.023 million (0.1202%) in 2010, 2015, and 2020, significantly higher than in the key areas: 0.778 million (0.1252%), 0.834 million (0.1278%), and 0.825 million (0.1212%). Similarly, for O_3_, the figures in the prevention and control areas were 0.096 million (0.01228%), 0.095 million (0.01243%), and 0.110 million (0.01316%), also higher than in the key areas: 0.0757 million (0.01218%), 0.078 million (0.01189%), and 0.090 million (0.01315%). Additionally, the Gini coefficients for PM_2.5_, O_3_, and overall health risks in the prevention and control areas were lower (0.182, 0.203, 0.284) compared to those in the key areas (0.207, 0.216, 0.292). This study provides a method for defining air pollution control regions based on health risks, offering significant insights for pollution zoning and prevention strategies

## 1. Introduction

With the rapid development of China’s economy, the pace of industrialization and urbanization has accelerated, and the problem of air pollution has become increasingly prominent [1,2]. Since 2013, China has successively issued three rounds of the “Ten Atmospheric Regulations” to carry out air pollution control with the strictest environmental protection system, achieving remarkable results over the past decade [3]. At the 2023 China Blue Sky Observation Forum, it was reported that in 2022, the annual average concentration of PM_2.5_ in China dropped to 29 μg/m^3^, falling below 30 μg/m^3^ for the first time.

Air pollution control has achieved remarkable results in reducing environmental pollution concentrations, but the health benefits remain relatively limited [4]. This is because the health risks associated with air pollution are influenced not only by pollutant concentrations in the environment but also by human activities in polluted areas [5]. Due to the spatial and temporal heterogeneity of the pollution and population distribution, the presence of pollutants does not necessarily reflect the distribution of health risks. Consequently, pollution control zones based on concentration levels may not coincide with areas of high health risk [6]. Some studies have shown that the national air pollution control priority zones do not always cover regions with the highest health risks [7]. In response, the national “Healthy China 2030” plan has emphasized the importance of identifying areas with high environmental health risks and assessing the impact of environmental pollution on public health.

Currently, air pollution monitoring technologies and the health risk assessment models related to air pollution have been relatively well developed. With the development of atmospheric remote sensing technology, it has become a primary means for obtaining high-spatiotemporal-resolution atmospheric pollutant concentrations. This technology constructs statistical models by integrating satellite remote sensing data, meteorological data, and geospatial data, achieving spatial resolutions down to the kilometer level [8,9,10]. Meanwhile, the Integrated Exposure Response (IER) model [11] and the Global Exposure Mortality Model (GEMM) [12] are commonly used for long-term health risk assessments of PM_2.5_ pollution, while log-linear models (LLMs) are frequently adopted for those of O_3_ pollution [13]. Although existing studies have conducted health risk assessments based on exposure–health response relationship models or identified pollution concentration control zones using remote sensing datasets and hotspot delineation methods, few have integrated these two approaches. As a result, the spatiotemporal variation in health risks is often overlooked in the delineation of control zones. Therefore, this study aims to delineate prevention and control zones based on health risk assessment results and assess their validity.

PM_2.5_ and O_3_ are two primary pollutants affecting air quality in China. While PM_2.5_ control measures are effective, the problem of O_3_ is becoming increasingly prominent [14]. Since 2015, national O_3_ pollution concentrations have shown a fluctuating upward trend [15]. China has entered a new stage of atmospheric compound pollution. The control of individual pollutants is no longer sufficient to address the current air pollution control needs in China [16,17]. On the one hand, there are differences in the areas of PM_2.5_ and O_3_ pollution. Therefore, the division of pollution prevention and control areas should consider both pollutants and their synergistic risks. On the other hand, PM_2.5_ and O_3_ share common precursors and have certain similarities [18]. The 14th Five-Year Plan also highlights the need to strengthen the coordinated control of multiple pollution sources [19]. Therefore, the coordinated prevention and control of PM_2.5_ and O_3_ pollution will become the focus of air pollution prevention and control in China in the future.

The purpose of this study is to address the lack of a regional delineation of prevention and control areas based on overall health risk assessments. On the basis of clarifying the spatial and temporal characteristics of health risks, considering the synergistic risks of the two pollutants, the PM_2.5_ and O_3_ synergistic risk prevention and control areas are divided to alleviate the health impact of the two major pollutants and realize the transformation from traditional pollution concentration control to health risk prevention and control and from focusing on the PM_2.5_ risk to focusing on the PM_2.5_ and O_3_ synergistic risk. In this study, health risk prevention and control areas (HAs) refer to regions identified based on integrated assessments of population exposure and pollutant-induced health risks, where intervention is prioritized due to high estimated health burdens. Among these, coordinated prevention and control areas are zones where PM_2.5_ and O_3_ present overlapping and synergistic health effects, requiring joint mitigation strategies due to their combined spatial impact and shared precursors. The research content to be carried out include a health risk assessment of air pollution, division of collaborative prevention and control areas, and comparative analysis of national key areas (KRs) and HAs. This paper explores the spatial and temporal changes in PM_2.5_ and O_3_ health risks in China, identifies the hotspots of health risks, and compares the HAs with the KRs from multiple perspectives, to provide an important scientific basis and reference for the joint prevention and control of atmospheric areas.

## 2. Materials and Methods

### 2.1. Study Data

**Figure 1:**
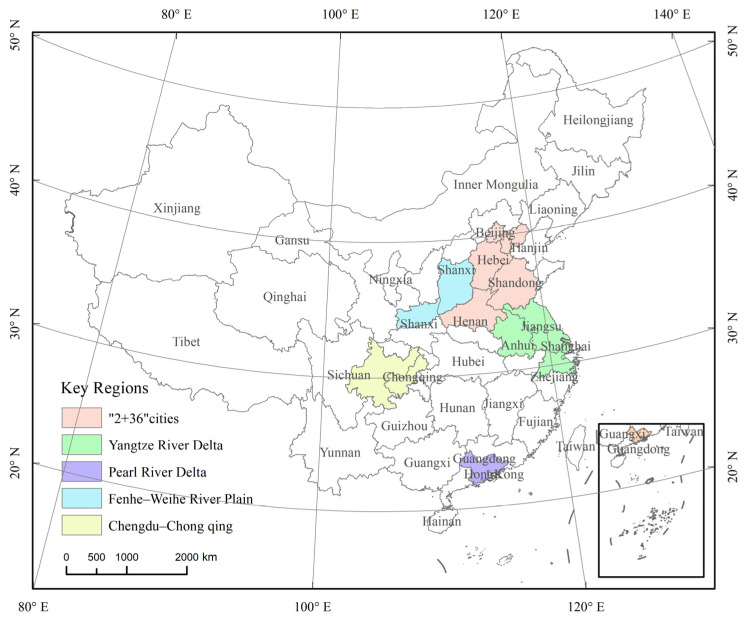
Study area and the location of key regions.

Mainland China is selected as the research area, and the key regions for air pollution prevention, as defined in the Action Plan for Continuously Improving Air Quality issued by the State Council in 2023, are selected as the key regions, including the Beijing–Tianjin–Hebei region and surrounding areas (“2 + 36” cities), the Yangtze River Delta (YRD) region, the Fenhe–Weihe River Plain region, the Pearl River Delta (PRD) region, and the Chengdu–Chongqing region. The study area and the five key regions are shown in Figure 1.

### 2.2. Methods

**Figure 2:**
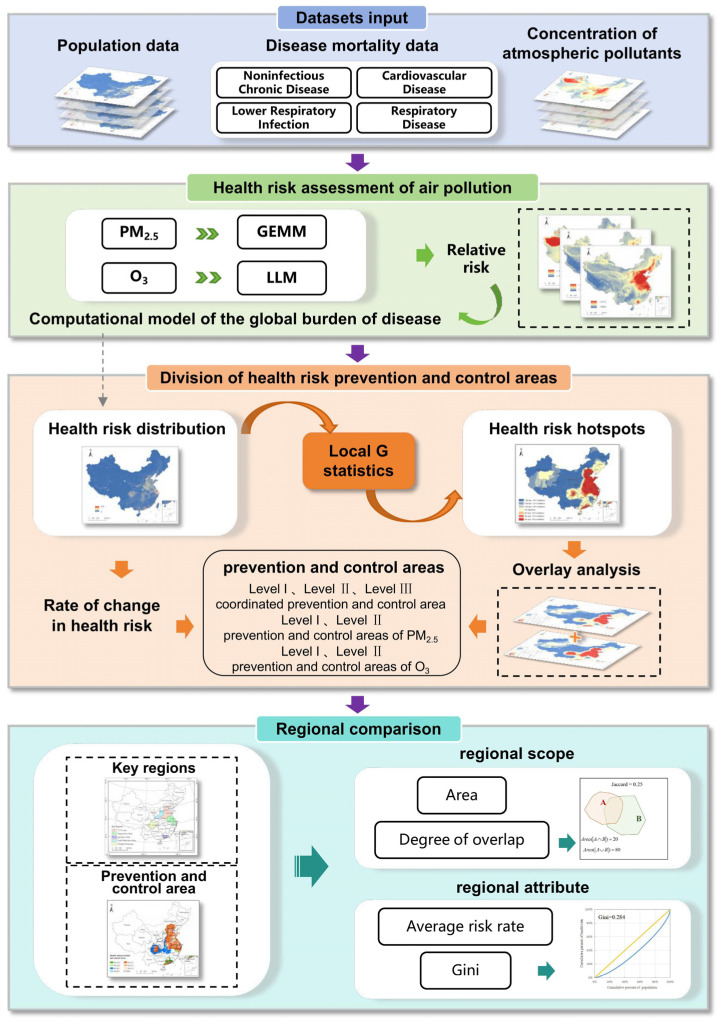
The flowchart of this study.

Different from traditional key regions for air pollution prevention and control, this study delineates health risk prevention and control areas from the perspective of reducing air pollution-related health risks, following a three-step approach. First, atmospheric pollutant concentration data, population statistics, and baseline mortality data for various diseases were utilized to estimate the health risks associated with PM_2.5_ and O₃, employing the GEMM and LLM, respectively. Next, spatial health risk hotspots were identified using the Local G statistic, and the HAs were delineated based on a spatial overlay analysis and rate of change in health risks. Finally, the KRs and HAs were compared in terms of their regional scope and attributes. The overall flowchart of this study is shown in Figure 2.

#### 2.2.1. Data

Atmospheric pollutant concentration data: This study utilized air quality data from the China Environmental Monitoring Center (https://air.cnemc.cn:18007/, (accessed on 27 March 2025)), where O_3_ was represented by the 90th percentile of the daily maximum 8 h moving average. In addition, gridded concentration datasets for PM_2.5_ (1 km resolution, 2000–2020) and O_3_ (10 km resolution, 2005–2020) were estimated using remote sensing-based statistical models. Specifically, satellite-derived aerosol optical depth, meteorological variables, and ground-based observations were integrated through a random forest regression algorithm to generate surface-level pollutant concentration maps. To ensure spatial consistency, O_3_ data were resampled to a 1 km resolution using bilinear interpolation prior to their use in the health risk assessment model [20].

Population data: derived from the WorldPop dataset (https://www.worldpop.org/, (accessed on 27 March 2025)). The WorldPop dataset is widely used in disease burden estimation, epidemic modeling, etc. The population data selected for this study are the population data with a 1 km resolution for different gender groups and age groups downloaded from the official website of the WorldPop dataset.

Disease baseline mortality data: derived from the official GDB website (https://ghdx.healthdata.org/gbd-results-tool, (accessed on 27 March 2025)). The mortality data attributed to non-communicable diseases, acute lower respiratory tract infections, respiratory diseases, and cardiovascular diseases for different gender and age groups from 2000 to 2019 in China were obtained. Due to the lack of disease benchmark mortality data in 2020, this study used 2019 data as a substitute.

#### 2.2.2. Health Risk Assessment of Air Pollution

In order to assess the health risk of air pollution in China, this study used the global burden of disease calculation model to estimate the health risks associated with PM_2.5_ and O_3_ in China, and the model formula is such as in Formula (1)

(1) $$M_{i,j,x}=K_{i,j,x}\times {POP}_{i,j}\times \left(1-\frac{1}{RR\left(C_{i,x}\right)}\right)$$

where $M_{i,j,x}$ refers to the number of health risks from disease *x* in age *i* and sex *j* exposed to a PM_2.5_ contaminated environment, where $M_{i,j,x}$ refers to *i* age group; *j* is the sex population of *x*; $K_{i,j,x}$ is the mortality rate of disease *x* in people with age *i* and sex *j*; ${POP}_{i,j}$ refers to the number of population in age *i*, sex *j*; and $RR(C_{i,x})$ is the relative risk of disease *x* in the *i* age group. The relative risk of PM_2.5_ is calculated by the GEMM [12], and the relative risk of O_3_ is calculated by an LLM [13]. To ensure clarity, central RR estimates were used, although the source models (GEMM and LLM) inherently incorporate uncertainty through confidence intervals. In this study, health risk specifically refers to the number of premature deaths attributable to air pollution [13,21].

#### 2.2.3. Division of Health Risk Prevention and Control Areas

##### Hotspot Area Identification

In this paper, Local G statistics are used to identify cold- and hotspots of health risks [22], in order to delineate atmospheric PM_2.5_ and O_3_ pollution health risk hotspots, with

(2) $$G_{i}^{*}=\frac{\sum_{j=1}^{n}w_{i,j}x_{i,j}-\bar{X}\sum_{j=1}^{n}w_{i,j}}{S\sqrt{\frac{\left[\sum_{j=1}^{n}w_{i,j}^{2}-{\left(\sum_{j=1}^{n}w_{i,j}\right)}^{2}\right]}{n-1}}}$$

(3) $$\bar{X}=\frac{\sum_{j=1}^{n}x_{j}}{n}$$

(4) $$S=\sqrt{\frac{\sum_{j=1}^{n}x_{j}^{2}}{n}-{\left(\bar{X}\right)}^{2}}$$

where *i* represents the central element, *j* is the element in the neighborhood, $x_{j}$ refers to the attribute value of element *j*, $w_{i,j}$ refers to the spatial weight between elements *i* and *j*, and *n* is the sum of the number of elements in the neighborhood.

##### Rules of Dividing Health Risk Prevention and Control Areas

Based on the results of the health risk hotspot analysis, health risk hotspot regions of atmospheric PM_2.5_ and O_3_ were extracted separately for overlay analysis. According to the pollutant objects, the whole country was initially divided into three types: PM_2.5_ prevention and control, O_3_ prevention and control, and PM_2.5_ and O_3_ coordinated prevention and control. On this basis, each area type was further subdivided into levels according to the rate of change in health risk. The rate of change in health risk is calculated as described in Formula (5). Based on this rate, each HA type was further subdivided into levels. Coordinated areas were classified into three levels: HA_CL1 (increasing risk for both pollutants), HA_CL2 (one increasing, one decreasing), and HA_CL3 (both decreasing). PM_2.5_ and O_3_ control areas were each divided into two levels: Level I for an increasing health risk (HA_PL1, HA_OL1) and Level II for a decreasing health risk (HA_PL2, HA_OL2). The detailed classification logic is summarized in Table 1.

(5) $$R{\left(X\right)}_{ij}=\frac{{M\left(X\right)}_{i}-{M\left(X\right)}_{j}}{{M\left(X\right)}_{j}}$$

where ${R(X)}_{i,j}$ is the rate of change in health risk from pollutant X between year i and year j, and ${M(X)}_{i}$, ${M(X)}_{j}$ represent the total health risks in years *i* and *j*, respectively.

#### 2.2.4. Regional Comparison

##### Quantitative Comparison of the Regional Scope

This article uses two indicators, area and degree of overlap, to quantitatively compare the delineated health risk prevention and control areas with the national five major pollution key areas. The degree of overlap is defined as follows: there are two regions *A* and *B*, and the ratio of the area of the intersection of *A* and *B* and the union area of *A* and *B* is the degree of overlap of the two regions, which is calculated by Formula (6).

(6) $$DOO\left(A,B\right)=\frac{Area\left(\left|A\cap B\right|\right)}{Area\left(\left|A\cup B\right|\right)}$$

where $DOO\left(A,B\right)$ is the overlap of regions *A* and *B*, $Area\left(\left|A\cap B\right|\right)$ is the area of the intersection of regions *A* and *B*, and $Area\left(\left|A\cup B\right|\right)$ is the area of the union of *A* and *B*.

##### Quantitative Comparison of the Regional Attributes

This paper uses the average risk rate and Gini coefficient to compare the divided prevention and control areas with the five key national anti-pollution areas to quantitatively compare their regional attributes and discuss the better solution of the problem of air pollution prevention and control.

The average risk rate (ARR) is the proportion of the population at health risk to the total population of the region, as calculated by Formula (7).

(7) $${ARR}_{i,j}=\frac{X_{i,j}}{Y_{j}}\times 100\%$$

where ${ARR}_{i,j}$ is the average risk rate of air pollutants *i* in region *j*, $X_{i,j}$ is the number of the population at health risk arising from air pollutants *i* in region *j*, and $Y_{j}$ is the total number of the population in region *j*.

In this paper, the Gini coefficient is used to evaluate the equality of air pollution health risk among regions. A smaller Gini coefficient indicates a more homogeneous distribution of health risks in the region, whereas a larger coefficient indicates inequality. The Gini coefficient is calculated as described in Formula (8).

(8) $$Gini=1-\sum_{i-1}^{n}\left(x_{i}-x_{i-1}\right)\left(y_{i}-y_{i-1}\right)$$

where *n* represents the number of counties divided, $x_{j}$ represents the cumulative percentage of health risk of an air pollutant, and $y_{j}$ represents the cumulative percentage of population in region *i*.

## 3. Results

### 3.1. Health Risk Assessment of Air Pollution

#### 3.1.1. Nationwide Health Risk Analysis

**Figure 3:**
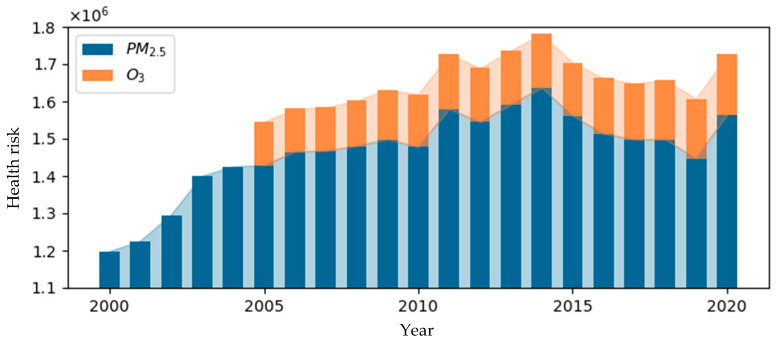
Annual health risk attributed to air pollution.

The results of health risks attributed to PM_2.5_ exposure from 2000 to 2020 are shown in Figure 3. Over this period, the average annual health risk was 1.463 million. The health risk fluctuated, rising from a low of 1.197 million in 2000 to 1.564 million in 2020, with the highest peak in 2014, reaching 1.636 million. After that, a gradual decline was observed, which may be attributed to the implementation of more comprehensive and synergistic air pollution control measures. Specifically, the in-depth implementation of the “Air Pollution Prevention and Control Action Plan” effectively reduced PM_2.5_ concentrations, directly leading to a decrease in health risks associated with PM_2.5_ exposure [24,25,26]. This trend highlights the significant effectiveness of China’s air pollution control policies in reducing public health burdens.

Similarly, the results of health risks attributed to O_3_ exposure from 2005 to 2020 are shown in Figure 3. During this period, the average annual health risk was 0.141 million. The health risk rose from 0.118 million in 2005 to 0.163 million in 2020, with the lowest recorded value being 0.115 million in 2006. Between 2008 and 2009, the health risk increased significantly, peaking at 116,000, and the growth rate slowed significantly after 2018. This change is speculated to be related to the first comprehensive management action targeting O_3_ precursor VOCs in 2018 [27].

#### 3.1.2. Health Risk Analysis of Key Regions

**Figure 4:**
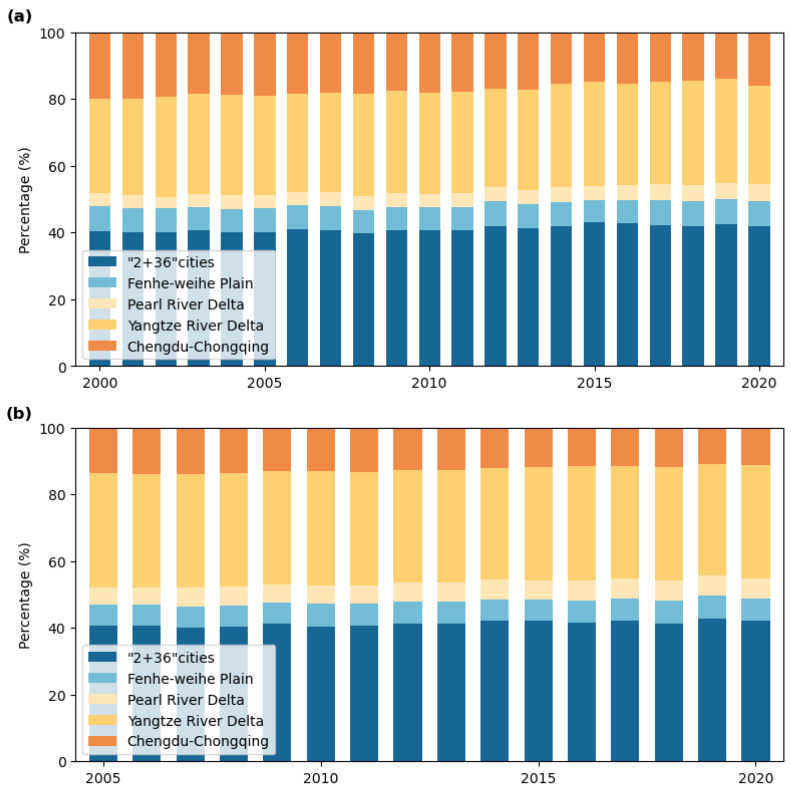
Health risk proportions across key regions: (**a**) PM_2.5_ related health risks; (**b**) O₃ related health risks.

The statistical results of health risks attributed to PM_2.5_ in the five key regions from 2000 to 2020 are shown in Figure 4a. Health risks from PM_2.5_ consistently accounted for over 50% of the national total each year, with an average annual proportion of 52.64%. The trend initially increased before declining, with the highest risk recorded in 2014 at 860,600. The “2 + 36” cities had the highest proportion, averaging 41.10%, followed by the YRD region at 30.11%, the Chengdu–Chongqing region at 17.33%, the Fenhe–Weihe Plain at 7.22%, and the PRD region at 4.25%.

The health risks attributed to O_3_ from 2005 to 2020 are shown in Figure 4b. O_3_-related health risks accounted for an average annual proportion of 53.64% of the national total, with a fluctuating upward trend, peaking at 89,500 in 2020. The distribution of O_3_ health risks across the five regions mirrored that of PM_2.5_, with the “2 + 36” cities, the YRD region, the Chengdu–Chongqing region, the Fenwei Plain, and the PRD region accounting for 41.32%, 33.84%, 12.60%, 6.54%, and 5.69%, respectively. Notably, the Chengdu–Chongqing region showed a significant reduction in its proportion of O_3_-related health risks compared to PM_2.5_.

### 3.2. Division and Analysis of Health Risk Prevention and Control Areas

#### 3.2.1. Division of Health Risk Prevention and Control Areas

**Figure 5:**
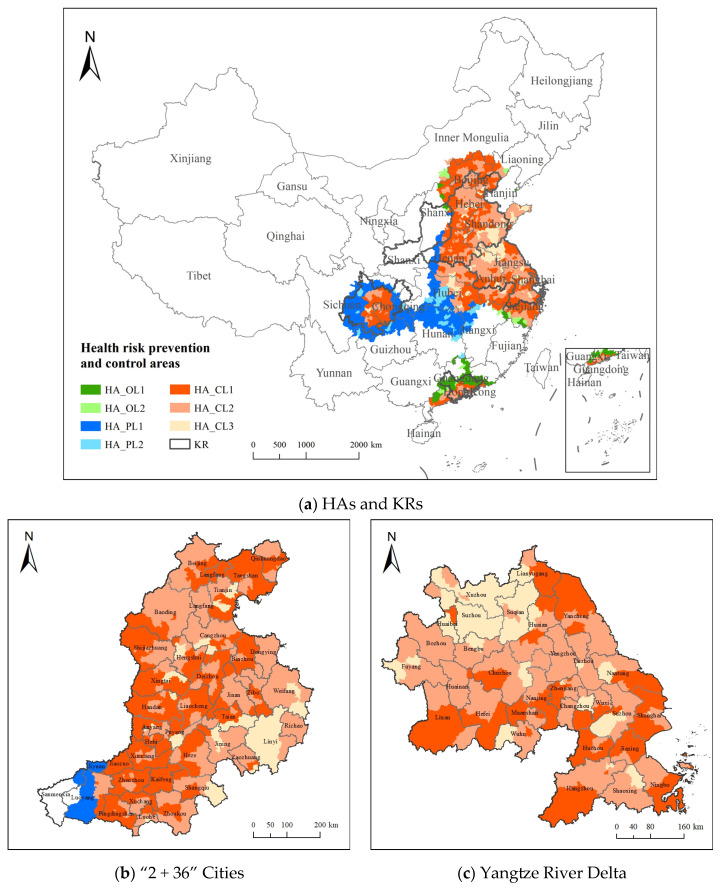

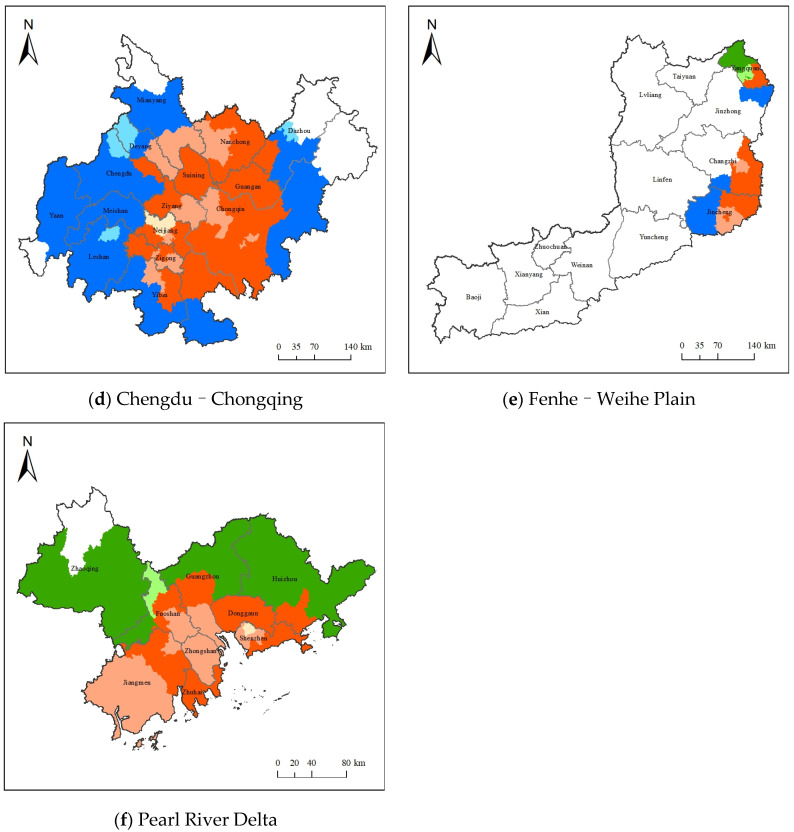
Prevention and control division in key regions.

Seven types of health risk prevention and control areas are divided according to the division rules. The results of health risk prevention and control areas in 2020 are shown in Figure 5a, and the statistics of health risk prevention and control areas are shown in Table 2. The health risk prevention and control area is 1,628,600 km^2^; HA_CL1 has the largest area of 52.75 km^2^, and HA_OL2 has the lowest area of 2.57 km^2^. Health risk in HAs accounted for 65.62% of the national total health risk, among which the health risk of HA_CL2 was the highest, accounting for 37.17% of the health risk in HAs, and the health risk of HA_OL2 was the lowest, accounting for 0.81% of the health risk in HAs.

The PM_2.5_ and O_3_ coordinated health risk prevention and control areas cover 988 county-level regions, with HA_CL1 comprising 46.92% of the regions, HA_CL2 accounting for 42.80%, and HA_CL3 making up 10.28%. These areas are primarily located in key regions such as the “2 + 36” cities, the YRD, and the Chengdu–Chongqing regions. HA_OL1 and HA_OL2 cover 44 and 17 regions, respectively, representing 73.58% and 26.42% of the area, mainly in Guangdong. HA_PL1 and HA_PL2 include 183 and 47 regions, with HA_PL2 comprising only 15.16%. PM_2.5_ prevention and control areas are concentrated in central and Southwestern China, while regions like Jiangxi and Shanxi face complex pollution control due to multiple pollutants.

The spatial distribution of prevention and control types in the key regions is shown in Figure 5b–f. The YRD region is within the coordinated prevention and control area, with HA_CL1, HA_CL2, and HA_CL3 accounting for 32.31%, 51.97%, and 15.72%, respectively. Most of the “2 + 36” cities region also falls within the coordinated health risk prevention and control areas, with HA_CL1, HA_CL2, and HA_CL3 accounting for 43.28%, 44.74%, and 9.05%, respectively. The Chengdu–Chongqing region is mostly within the coordinated prevention and control area and HA_PL1. In the Fenhe–Weihe Plain, 86.52% is outside the health risk prevention and control areas, while the PRD region is mainly located within coordinated health risk prevention and control areas and HA_OL1.

#### 3.2.2. Quantitative Comparison of Scope Between KRs and HAs

The quantitative comparison results of the health risk prevention and control areas and key regions are shown in Table 3. The table compares the areas of KRs, HAs, and the PM_2.5_ prevention and control section (RP_P) and O_3_ prevention and control section (RP_O) for the years 2010, 2015, and 2020. RP_P includes parts of HA_PL1, HA_PL2, HA_CL1, HA_CL2, and HA_CL3, while RP_O includes parts of HA_OL1, HA_OL2, HA_CL1, HA_CL2, and HA_CL3 within the HAs. The table also presents the regional overlap between KRs and HAs, as well as between KRs and the RP_P and the RP_O. The results indicate that the area of HAs is larger than that of KRs, and that the spatial overlap between KRs and HAs, as well as between KRs and RP_P and RP_O, is approximately 0.5. This suggests that national key regions currently cover only about half of the areas with high health risks, revealing a certain degree of spatial mismatch between existing policy-defined zones and health risk distributions. Notably, the overlap between RP_P and KRs is relatively higher than that between RP_O and KRs, indicating that recent control efforts have been more focused on PM_2.5_.

#### 3.2.3. Quantitative Comparison of Attributes Between KRs and HAs

The quantitative comparison results of the attributes of health risk prevention and control areas and key regions are shown in Table 4. The table compares the health risk values of KRs and HAs during 2010, 2015, and 2020, as well as the average health risk attributed to PM_2.5_ in HA_P and KRs, and the average health risk attributed to O_3_ in HA_O and KRs. The results show that, regardless of whether the health risks are attributed to PM_2.5_ or O_3_, the health risks in HAs are higher than in KRs, with the average health risk of all parts of HAs generally being greater than that of KRs. This indicates that the delineated areas are more effective than KRs in the prevention and control of health risks.

**Figure 6:**
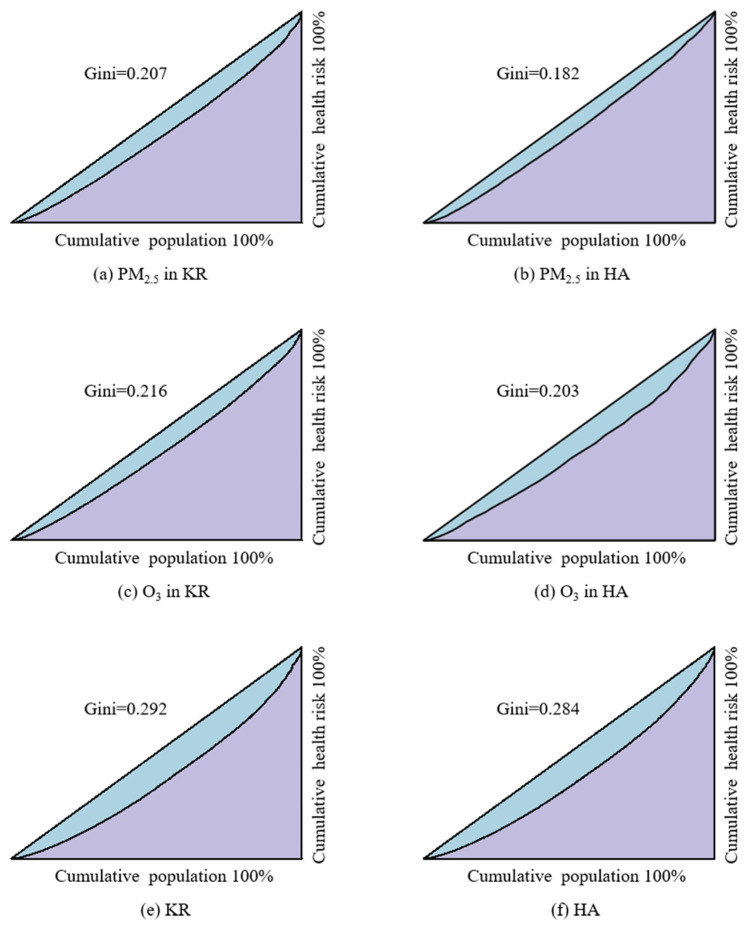
Gini coefficient of health risk.

The health risk distribution of KRs and HAs was analyzed using the Lorenz curve and Gini coefficient. For HAs, HA_P was selected to calculate the Gini coefficient of health risk attributed to PM_2.5_, and HA_O was selected to calculate the Gini coefficient of health risk attributed to O_3_. The Gini coefficient for total health risk was calculated for collaborative prevention and control. The results showed that the Gini coefficient for health risk attributed to PM_2.5_ was 0.207 in KRs (Figure 6a) and 0.182 in HA_P (Figure 6b), for O_3_ it was 0.216 in KRs (Figure 6c) and 0.203 in HA_O (Figure 6d), and for total health risk, it was 0.292 in KRs (Figure 6e) and 0.284 in HAs (Figure 6f). The Gini coefficient for the corresponding health risk of pollutants in KRs is higher than that in HAs, indicating that the divided health risk prevention and control areas are more equitable and reasonable compared to the internal health risk distribution in KRs. Furthermore, the classification and control of HAs based on air pollutant types make regional air pollution control more targeted. Classified prevention and control contribute to more efficient regional joint efforts in air pollution control.

### 3.3. Spatiotemporal Variations in Health Risk Prevention and Control Areas

#### 3.3.1. Health Risk Variations in Health Risk Prevention and Control Areas

**Figure 7:**
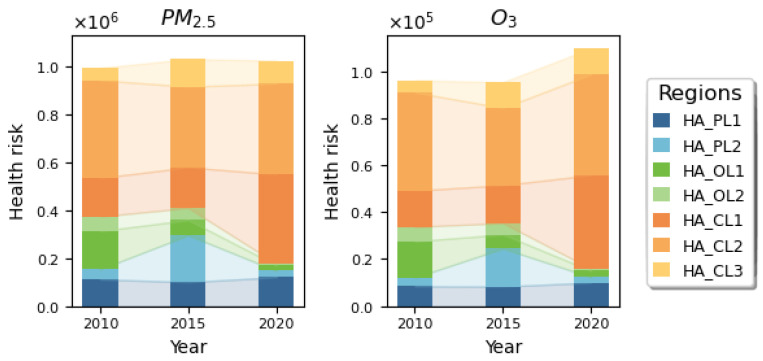
Health risk changes in the health risk prevention and control areas.

The changes in PM_2.5_ and O_3_ health risks in the health risk prevention and control areas in 2010, 2015, and 2020 are shown in Figure 7. The results indicate that the average health risk attributed to PM_2.5_ in the prevention and control areas was 1,015,273, while the average health risk attributed to O_3_ was 100,345, accounting for more than 65% of the national health risk. For PM_2.5_ pollution, the highest health risk in the prevention and control areas occurred in 2015, with 1,030,230 people affected, while for O_3_ pollution, the highest health risk in the prevention and control areas occurred in 2020, with 109,702 people affected. The proportion of health risks from O_3_ in the main prevention and control areas was the lowest and decreased year by year. The proportion of health risks in the collaborative control areas exceeded 85% in 2020, confirming that China has entered a new stage of atmospheric composite pollution control. The coordinated prevention and control of PM_2.5_ and O_3_ pollution will become the focus of future atmospheric pollution control efforts in the country.

#### 3.3.2. Scope of Variation in Health Risk Prevention and Control Areas

**Figure 8:**
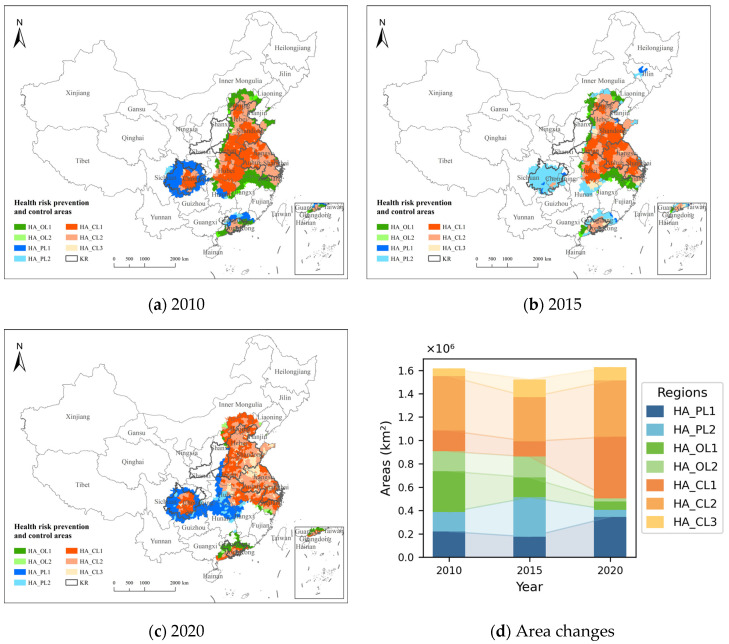
Division of health risk prevention and control areas.

The changes in the HA boundaries for 2010, 2015, and 2020 are shown in Figure 8a–c, and the area changes for the seven categories of HAs in 2010, 2015, and 2020 are shown in Figure 8d. The results indicate that, in 2020, the largest area was 1,628,653 km^2^, while the smallest area in 2015 was 1,523,078 km^2^. The cooperative prevention and control areas had the largest proportion, with a significant increase in area. In 2020, the area reached 1,124,174 km^2^, accounting for 69.02% of the total area. Among these, HA_CL1 saw the most significant increase, with an area growth of 349,109 km^2^ in 2020 compared to 2010. HA_OL2 had the smallest proportion, and the O_3_ prevention and control area decreased year by year, from 519,200 km^2^ in 2010 to 97,372 km^2^ in 2020. As a result, the proportion of the total prevention and control area decreased from 32.12% to 5.99%. The prevention and control area dominated by PM_2.5_ was the largest in 2015, covering 514,006 km^2^ or 33.75% of the total area. Among these, the proportion of HA_PL1 increased significantly, from 57.00% in 2010 to 84.84% in 2020. The northwest and western regions of the HAs have expanded, incorporating more areas in the Shaanxi, Inner Mongolia, Hunan, Guizhou, and Chongqing provinces, while the southern regions of the HAs have shrunk. The areas in the Jiangxi, Fujian, and Zhejiang provinces have gradually decreased. The scope of coordinated prevention and control areas has expanded to the north, with central China predominantly consisting of these areas. The level of prevention and control areas has gradually decreased, with the PM_2.5_ prevention and control areas in Hunan and Hubei increasing, while those in Guangdong have decreased. By 2020, only the coordinated prevention and control areas and O_3_ prevention and control areas remained.

## 4. Discussion

Based on the health risk assessment model of air pollution, this study proposes a method to divide the coordinated prevention and control area of air pollution from the perspective of regional overall health risk prevention and control, and it delineates seven types of air pollution prevention and control areas. Since the implementation of the “Air Pollution Prevention and Control Action Plan” in 2013, China has made significant progress in air pollution prevention and control, with continuous improvement in national air quality. From 2013 to 2021, the PM_2.5_ concentration decreased from 48.0 μg/m^3^ to 27.3 μg/m^3^, with more significant reductions in key regions such as Beijing–Tianjin–Hebei, the Yangtze River Delta, and the Pearl River Delta [28]. The division of the five major key control regions in China is primarily based on the severity of pollution and administrative divisions, facilitating regional joint prevention and control [29]. However, from the perspective of health risk prevention and control, this division still has certain limitations.

Compared with the research that only considers a single pollutant or simply divides the prevention and control area according to the type of pollutant, the prevention and control area divided in this study considers the synergistic risk of PM_2.5_ and O_3_, and it further subdivides the prevention and control level of different pollutant prevention and control areas according to the annual change rate of health risk, so that the regional air pollution control is more targeted and can better meet the formulation and implementation of the national precise prevention and control strategy at this stage. From the perspective of risk prevention and control, in the comparison with the five key areas of the country, the prevention and control areas identified in this study show advantages of high values of overall health risk and average risk rate and small values of the Gini coefficient index. It means the division is more objective and reasonable, which can be helpful for the country to take accurate action in atmospheric prevention and control.

This study identifies high-risk areas for health impacts from PM_2.5_ and O_3_ pollution, driven by the interplay of high pollutant concentrations, adverse weather, regional transmission, and factors like population density, industrial emissions, and energy structure. Differences in pollution control types across regions reflect these factors. For example, the Yangtze River Delta and the “2 + 36” cities are densely populated and industrialized, with high emissions of PM_2.5_ and O_3_ precursors (e.g., VOCs, NOx), primarily focused on integrated control. The Pearl River Delta reduced PM_2.5_ early through industrial restructuring but now faces increasing O_3_ pollution, influenced by local weather conditions, making O_3_ the focus of control. In the Chengdu–Chongqing region, pollution dispersion is limited by basin topography, resulting in severe PM_2.5_ pollution and stable O_3_ levels, leading to a mixed control approach. The Fenwei Plain has seen improvements in both pollutants, with a low population density contributing to a smaller proportion of high-risk areas.

The delineation of health risk prevention and control areas provides targeted strategies for air pollution management and risk avoidance for both the government and the public. For the government, different types of control areas require specific measures: in coordinated prevention and control areas, the focus should be on the synergistic control of O_3_ and PM_2.5_, based on their regional and seasonal correlations [24,25]; in PM_2.5_ prevention and control areas, emphasis should be placed on reducing both primary and secondary PM_2.5_ emissions, while promoting green transformation in industry and transportation [28]; in O_3_ prevention and control areas, controlling VOCs and NOx precursors and updating species-specific emission inventories are key [29]. For the public, raising environmental health awareness is critical [30,31,32]. Residents in different control areas have distinct priorities, and even within the same area, factors such as gender, age, and health status influence the sensitivity to pollution concentrations [33]. Daily travel is the main exposure route to air pollution [34], with travel choices closely tied to “where to go” and “how to go”. The public should use health risk assessments and monitoring information provided by the government, combined with personal health conditions, to choose healthier travel routes and minimize exposure to outdoor air pollution [34,35,36].

Against the background of global climate change, the increasing frequency of multiple environmental pollution and extreme weather events has made health risk prevention and control more difficult than considering only a single environmental factor. The method proposed in this study for dividing prevention and control zones based on air pollution health risks can be applied in similar fields. For example, concurrent heatwaves and ozone always occur in summer [30,31], and such composite events can increase the risk of ischemic stroke and chronic obstructive pulmonary disease [32,33]. In this situation, further measures can also be considered, such as the establishment of an early-warning and forecasting mechanism for health risks, optimizing the allocation of regional medical facilities and services, and adjusting urban forms. For example, in the case of urban air pollution emergencies, an immediate emergency medical response mechanism should be activated, with the establishment of dedicated medical rescue teams and enhanced scheduling arrangements for clinics related to respiratory and other diseases, to face the impact of public health emergencies in an orderly and composed manner [34]. In terms of medical infrastructure allocation, it is crucial to ensure even and accessible coverage of health resources within the region, guiding the allocation of spatial resources towards vulnerable groups to achieve health equity [35]. Regarding urban forms, it is necessary to reasonably plan and control the structure and layout of urban and rural construction land, implement stricter pollutant emission standards and total emission control indicators in optimized development areas, and establish a low-carbon-oriented territorial spatial system [36].

This study also has several limitations. First, the limitations as to the data’s timeliness may place some constraints on the interpretation of the findings. Although the long-term dataset enabled us to reveal the spatial–temporal patterns of health risks associated with PM_2.5_ and O_3_, the analysis is constrained by the availability of national health statistics. Due to the lack of validated data beyond 2020, the most recent year of analysis is limited to 2020, which may impact the timeliness of the findings. Future studies will incorporate more updated datasets as they become available. Secondly, the model has not fully accounted for spatial heterogeneity in the exposure–response relationships. While this study considered differences in the exposure–response relationships across age and sex groups, it did not systematically address variations in the exposure–response relationships across different regions [37]. The relationship between air pollution and mortality rates differs between urban and rural areas, influenced by economic, social, and environmental factors [38], and these disparities remain an important direction for future refinement of the model. Third, the scope of pollutants included in the assessment remains limited. The health risk assessment in this study was based on two major pollutants, PM_2.5_ and O_3_,while other harmful atmospheric components such as NO_2_, SO_2_, and CO were not included [39]. A more comprehensive assessment integrating multiple pollutants would better reflect the complex health burden posed by atmospheric pollution.

## 5. Conclusions

In response to the increasingly severe air pollution health issues in China and the fact that most existing studies only divide air pollution control zones based on pollutant concentrations, while overlooking environmental exposure processes, this study proposes a method for delineating control zones from the perspective of overall regional health risk prevention and control. Based on air pollutant concentration data, combined with health risk assessment models and spatial clustering methods, this study clarifies the spatiotemporal distribution patterns of health risks associated with PM_2.5_ and O_3_. To effectively mitigate the health problems caused by these two major pollutants, it defines coordinated prevention and control zones for both pollutants, better supporting the national strategy for the joint control of PM_2.5_ and O_3_. Compared to the national key regions, the prevention and control zones delineated in this study outperform the key areas in terms of health risk values, average risk rates, and Gini coefficients. The air pollution control zones identified in this study are more objective and reasonable, with a lower internal health risk inequality, better reflecting the concept of health equity. This provides an important basis and reference for formulating future air pollution control policies, conducting regional joint prevention and control efforts, and managing regional environmental quality. Future research should be expanded in several key directions. First, examining urban–rural disparities in pollution exposure and healthcare access would enable a more precise identification of spatial health inequalities. Second, accounting for population aging trends is crucial to better assess exposure vulnerability among high-risk groups like the elderly [40,41]. Third, incorporating data on residents’ daily mobility patterns and dynamic exposure characteristics would yield more realistic individual risk assessments. Finally, studies should move beyond single-pollutant analyses to investigate the complex interactions between multiple pollutants and extreme weather events.

## Figures and Tables

**Table 1:** Basis for dividing health risk prevention and control zones.

Area Type	Pollutants Objects	Prevention and Control Level	Constraint Condition
HA_CL1	PM_2.5_ and O_3_ coordinated prevention and control	Level I	[R(PM_2.5_) ≥ 0] ∩ [R(O_3_) ≥ 0]
HA_CL2		Level II	{[R(PM_2.5_) ≥ 0] ∩ [R(O_3_) < 0]} [23]∪{[R(PM_2.5_) < 0] ∩ [R(O_3_) ≥ 0]}
HA_CL3		Level III	[R(PM_2.5_) ≥ 0] ∩ [R(O_3_) ≥ 0]
HA_PL1	PM_2.5_ prevention and control	Level I	R(PM_2.5_) ≥ 0
HA_PL2		Level II	R(PM_2.5_) < 0
HA_OL1	O_3_ prevention and control	Level I	R(O_3_) ≥ 0
HA_OL2		Level II	R(O_3_) < 0

**Table 2:** Statistics of health risk prevention and control areas.

Type of Prevention and Control Area	Area (10^4^ km^2^)	County-Level Administrative Districts	PM_2.5_ Health Risk (10^4^ People)
HA_PL1	34.54	183	1.21
HA_PL2	6.17	47	0.27
HA_OL1	7.16	44	0.22
HA_OL2	2.57	17	0.08
HA_CL1	52.75	454	3.71
HA_CL2	48.12	435	3.78

**Table 3:** Regional scope quantitative comparison.

Year	Area (10^4^ km^2^)				Degree of Overlap		
	KR	HA	HA_P	HA_O	HA	HA_P	HA_O
2010	104.33	161.64	109.72	122.98	0.47	0.51	0.44
2015		152.31	117.60	100.91	0.49	0.53	0.42
2020		162.87	153.13	122.15	0.48	0.48	0.49

**Table 4:** Quantitative comparison of regional attributes.

Year	Health Risk (10^4^ People)				Average risk Rate (%)			
	PM_2.5_		O_3_		PM_2.5_		O_3_	
	KR	HA	KR	HA	KR	HA_P	KR	HA_O
2010	77.81	99.26	7.57	9.61	0.12520	0.12861	0.01218	0.01228
2015	83.40	103.02	7.76	9.52	0.12788	0.12829	0.01189	0.01243
2020	82.45	102.30	8.95	10.97	0.12122	0.12020	0.01315	0.01316

## Data Availability

Not applicable.
